# The complete mitochondrial genome of *Cerion uva uva* (Gastropoda: Panpulmonata: Stylommatophora: Cerionidae)

**DOI:** 10.1080/23802359.2017.1303343

**Published:** 2017-03-21

**Authors:** M. G. Harasewych, Vanessa L. González, Amanda M. Windsor, Margaret Halloran

**Affiliations:** a Department of Invertebrate Zoology, National Museum of Natural History, Smithsonian Institution, Washington, DC, USA;; b Laboratory of Analytical Biology, National Museum of Natural History, Smithsonian Institution, Washington, DC, USA

**Keywords:** Curaçao, mitogenome, Mollusca

## Abstract

We report the complete mitochondrial genome sequence of *Cerion uva uva* (Linnaeus 1758), the type species of the type genus of the family Cerionidae. The mitogenome is 15,043 bp in length, has a base composition of A (28.3%), T (34.4%), C (17.3%) and G (20.0%), and contains 13 protein-coding genes, 2 ribosomal RNA genes, as well as 22 transfer RNA genes. Gene order is the same as in *Cerion incanum* (Leidy 1851), but differs from those of all other Panpulmonata. This is the second mitochondrial genome sequenced within the family Cerionidae and will contribute to the assessment of the phylogeography of this family throughout the islands of the tropical western Atlantic.

Land snails of the *Cerion uva* complex are endemic to the islands of Aruba, Curaçao, and Bonaire (Baker 1924; De Vries 1974; Gould 1984). *Cerion uva uva*, the nominotypical subspecies of the type species of the type genus of the family Cerionidae, was isolated in eastern Curaçao during Pleistocene interglacial high sea-level stands (Wagenaar Hummelinck 1990), and was introduced to Aruba by humans within the past 800 years (Harasewych 2015). Recent phylogenetic analyses (Harasewych et al. 2011, 2015) have shown *Cerion uva* to be the most basal, and *Cerion incanum* (from the Florida Keys) to be among the most derived of the Cerionidae inhabiting the islands of the tropical western Atlantic.

We sequenced the complete mitochondrial genome of *Cerion uva uva* (GenBank accession number KY124261) using genomic DNA from a specimen (USNM 1153961L) collected at the type locality for this taxon (Schaarlo, Curaçao, 12° 6.42′ N, 68° 55.47′ W). Both partial COI and 16S sequences of this specimen have previously been published (Harasewych 2015). Extracted DNA was quantified using a Qubit dsDNA BR Assay Kit (ThermoFisher, Pittsburgh, PA). After quantification, DNA was sonicated (Qsonica Q800R2, QSonica, Newtown, CT) and libraries were prepared using the NEBNext^®^ Ultra™ DNA Library Prep Kit for Illumina^®^ along with the NEBNext Multiplex Oligos for Illumina. Size selection of adaptor-ligated libraries (300–700 bp) was performed using the Pippin Prep. The Agilent TapeStation (Agilent, Santa Clara, CA) was used to validate library size selection. Libraries were quantified using qPCR (ViAA 7) to ensure generation of adaptor-ligated libraries. A 4nM library concentration was denatured for clonal amplification and sequencing on the Illumina Miseq, run at the Smithsonian’s Laboratories of Analytical Biology.

We obtained 4,808,211 sequences ranging in length from 35 to 301 bp. The mitogenome was assembled using the ‘map to reference’ feature of Geneious v. 9.1.6 (Biomatters, Newark, NJ) with the previously published (Harasewych 2015) partial COI sequence [Haplotype 57, GenBank KJ624976] from this specimen as the initial reference sequence. The mitogenome was independently reconstructed using the previously published partial 16S sequence [Haplotype 117, GenBank KJ636147] as the initial reference sequence. The two independently constructed mitogenomes were identical. A total of 10,398 reads mapped to the mitochondrial genome. Coverage ranged from 8× to 94× per site (46.5 ± 14.1). Mitochondrial elements were annotated using MITOS (Bernt et al. 2013), ARWEN (Laslett & Canbäck 2008) and the ORF finder in Geneious.

The mitochondrial genome of *Cerion uva uva* is a circular molecule consisting of 15,043 bp (134 bp shorter than *C. incanum*), with a base composition of A (28.3%), T (34.4%), C (17.3%) and G (20.0%). As in other pulmonates (Figure 1) (González et al. 2016), it contains 13 protein-coding genes, 2 ribosomal RNA genes, as well as 22 transfer RNA genes. Gene order is identical to that of *Cerion incanum* (González et al. 2016) (Figure 1), but differs from that of other presently known Panpulmonata. This is the second mitochondrial genome sequenced within the family Cerionidae and will contribute to the assessment of the phylogeography of this family throughout the islands of the tropical western Atlantic.

**Figure 1.**
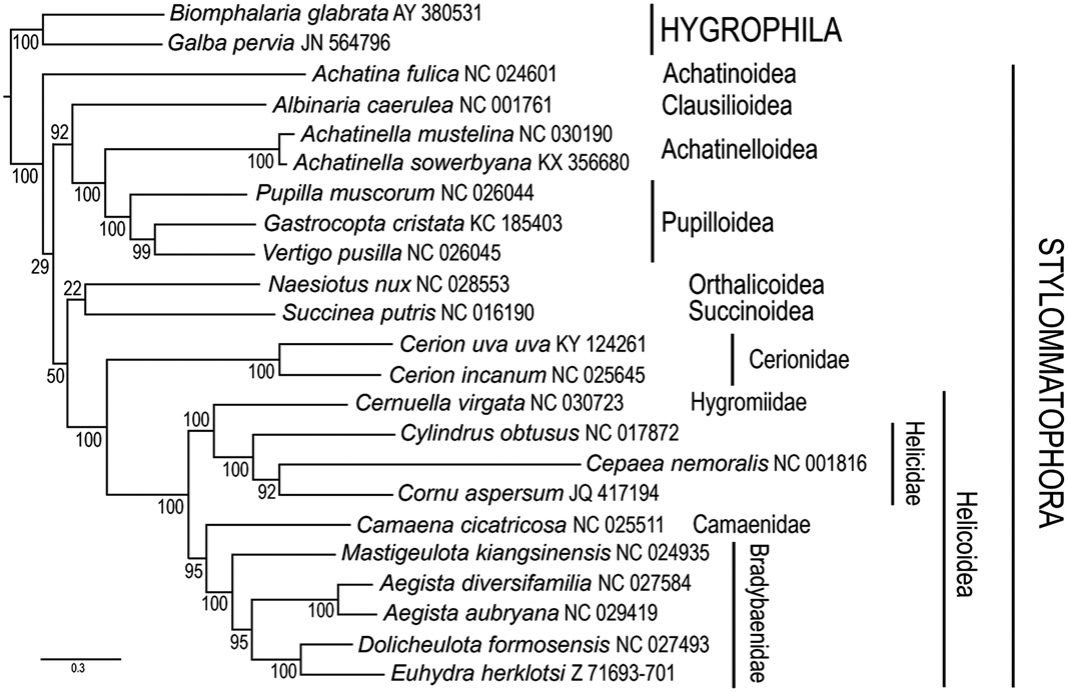
Placement of *Cerion uva uva* among Stylommatophoran snails. Amino-acid sequences of protein-coding genes were individually aligned using the L-INS-i option with default parameters of the MAFFT v. 7 aligner (Katoh & Stanley 2013). Nucleotide alignments for individual protein-coding genes were obtained according to their amino-acid alignments using PAL2NAL (Suyama et al. 2006). Ribosomal genes were individually aligned using MAFFT (Q-INS-i option) (Katoh & Toh 2008). Individual nucleotide gene alignments were filtered using Gblocks (Talavera & Castresana 2007) with default parameters, allowing gaps in all positions. These were subsequently concatenated, leading to alignments with 9785 nucleotide positions for protein-coding genes and 1282 positions for the rRNA genes. Maximum likelihood analyses were performed using RAxML v. 8 (Stamatakis 2014). The general time reversible (GTR) model of nucleotide evolution was used. Maximum likelihood analyses consisted of 1000 independent tree searches and bootstrap runs. All analyses were run on the Smithsonian Institution’s high-performance computing cluster (SI/HPC). The resulting trees show similar relationships to previous studies (Gonzalez et al. 2016).
